# Chronic kidney disease in patients with normal eGFR at baseline: results from EuroSIDA

**DOI:** 10.1186/1758-2652-13-S4-P82

**Published:** 2010-11-08

**Authors:** L Ryom, A Mocroft, P Reiss, B Ledergerber, S De Wit, D Duiculescu, AD Monforte, M Murphy, JD Lundgren, O Kirk

**Affiliations:** 1 Copenhagen HIV Programme, University of Copenhagen, Faculty of Health Sciences, Dept. of infectious diseases, Copenhagen, Denmark; 2 University College London, Medical School, Royal Free Campus, London, UK; 3 Academisch Ziekenhuis bij de Universiteit van Amsterdam, Amsterdam, Netherlands; 4 University Hospital Zurich, Division of Infectious Diseases, dept. of medicine, Zurich, Switzerland; 5 CHU Saint-Pierre, Department of Infectious Diseases, Brussels, Belgium; 6 Dr. Victor Babes Hospital, Spitalul de Boli Infectioase si Tropical, Bucuresti, Romania; 7 Ospedale San Paulo, Dipartimento di Medicina, Chururgia e Odontoiatria, Clinica delle Malattie Infettive e Tropicali, Milano, Italy; 8 Royal London hospital, Grahame Hayton Unit, Ambrose King Center, London, UK

## Background

Chronic kidney disease (CKD) is an emerging co-morbidity among HIV patients. Recent EuroSIDA analyses identified CKD risk factors including hypertension, diabetes, hepatitis C, age>50, low CD4 count, prior AIDS events and cumulative exposure to certain antiretrovirals (ARVs; tenofovir, indinavir atazanavir and probably lopinavir/ritonavir).

## Objectives

We aimed to extend our previous findings by estimating the CKD incidence among patients with normal kidney function at baseline with and without other risk factors, in order to disentangle if ARVs also pose a risk to patients with normal kidney function , and not only to those with pre-existing impairment.

## Methods

Cockcroft-Gault equation standardised for body surface was used to estimate Glomerular filtration rate (eGFR, ml/min/1,73m2). Patients with baseline eGFR> 90 were included. Baseline was defined as the first eGFR assessment after 01.01.2004. CKD was defined as 2 consecutive eGFR<60 (>3 months apart). Follow-up was from baseline until CKD or last eGFR. Unadjusted incidence rates (IR) are presented per 100 PYFU and stratified by cumulative ARV exposure.

## Results

**Figure 1:**
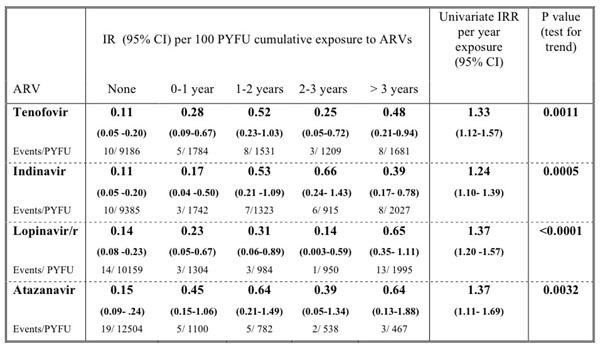


4824 patients had baseline eGFR>90. They were predominantly white (86.4%), male (74.4%) infected via homosexual contact (41.4%). At baseline 17.6% had hypertension, 3.7% diabetes and 24.1% hepatitis C. Median age was 40 (IQR: 34.6-45.1) years, and median CD4 count 446 (300-640) cells/mm3. During 15391 PYFU and a median follow-up of 41 (IQR 21-56) months, 34 (0,7%) developed CKD (IR 0.22, 95%CI 0.15-0.30). Among patients without traditional risk factors, 7 patients developed CKD during 8076 PYFU (IR 0.09 95%CI 0.04-1.18). In unadjusted analyses CKD incidences increased with increasing cumulative ARV exposure for the ARVs tested (test for trend significant for all drugs investigated) (Figure 1).

## Conclusions

This study of almost 5000 patients and a median follow-up >3 years demonstrates that CKD development from normal kidney function was infrequent. The IR was higher in patients with renal risk factors and those cumulative exposed to the ARVs investigated in unadjusted models. This suggests that ARVs might also pose a risk in patients with normal kidney function. Adjusted analyses were not possible due to low IR. Future studies with substantially larger size and longer follow up are needed to reproduce the findings in adjusted models, determine the role of cumulative exposure to individual ARVs and investigate the clinical implications.

